# A gapmer antisense oligonucleotide targeting *SRRM4* is a novel therapeutic medicine for lung cancer

**DOI:** 10.1038/s41598-019-43100-1

**Published:** 2019-05-20

**Authors:** Masahito Shimojo, Yuuya Kasahara, Masaki Inoue, Shin-ichi Tsunoda, Yoshie Shudo, Takayasu Kurata, Satoshi Obika

**Affiliations:** 1 Graduate School of Pharmaceutical Sciences, Osaka University, 1-6 Yamadaoka, Suita, Osaka 565-0871 Japan; 2 National Institutes of Biomedical Innovation, Health and Nutrition (NIBIOHN), 7-6-8 Saito-Asagi, Ibaraki, Osaka 567-0085 Japan; 3 Kansai Medical University, 2-5-1 Shin-machi, Hirakata, Osaka 573-1010 Japan; 4 Present Address: The Faculty of Pharmaceutical Sciences, Kobe Gakuin University, 1-1-3 Minatojima, Chuo-ku, Kobe, Hyogo, 650-8586 Japan

**Keywords:** Drug development, Small-cell lung cancer

## Abstract

Small cell lung cancer (SCLC) is the most aggressive neuroendocrine phenotype of the deadliest human lung cancers. However the therapeutic landscape for SCLC has not changed in over 30 years. Effective treatment and prognosis are needed to combat this aggressive cancer. Herein we report that Ser/Arg repetitive matrix 4 (SRRM4), a splicing activator, is abnormally expressed at high levels in SCLC and thus is a potential therapeutic target. We screened an effective gapmer antisense oligonucleotide (gASO) targeting SRRM4 *in vitro* which led to cell death of SCLC. Our gASO, which is stabilized by containing artificial nucleotides, effectively represses SRRM4 mRNA. We found that our gASO repressed SRRM4 synthesis leading to a dramatic tumor reduction in a lung cancer mouse model. We also analyzed miRNA microarray and found that the miR-4516 is abnormally increased in exosomes in the blood of SCLC patients. Treating with gASO suppressed tumors in the SCLC model mouse concurrently reduced plasma miR-4516. In conclusion this study reports that administration of an SRRM4-targeted gASO coupled with a novel miRNA diagnostic methodology represents a potential breakthrough in the therapeutic treatment of high mortality SCLC.

## Introduction

Lung cancer is a major cause of cancer-related deaths worldwide, leading to over a million deaths a year^1^. Lung cancer is histologically classified into two main types, small-cell lung cancer (SCLC) and non-small cell lung cancer (NSCLC)^2^. The diagnosis for SCLC or NSCLC as well as a mix of the two is difficult because of lung cancer heterogeneity. SCLC is a high-grade neuroendocrine carcinoma exhibiting as a highly aggressive subtype of cancer^3^. Characteristics of SCLC are distant metastatic and lethal tumors that cause the death of over 1.6 million people worldwide annually^4^. Initial treatment with anti-cancer drugs effectively suppresses SCLC, however relapse to forms resistant to therapy frequently occur leading to death. NSCLC patients also harbor SCLC during the onset of therapeutic treatment, making poor prognosis. Molecular targeted medicine for NSCLC has been developed and raised the success rate for therapy. Clinical drugs targeting angiogenesis, DNA repair or apoptosis has been developed and tested for SCLC treatment, however the therapeutic landscape for aggressive SCLC has not changed for over 30 years. Thus, SCLC is categorized as a recalcitrant cancer^5^ and novel therapeutics and prognostics are needed.

Tumor heterogeneity between SCLC and NSCLC was reported to be caused by several pathways^6–8^. One of the master molecules is an RE1-Silencing Transcription factor (REST, also known as Neuron-Restrictive Silencer Factor, NRSF)^9^. REST acts as a major tumor suppressor through its gene-silencing action. It functions by binding to an RE1 element in its target genes, many of which are neuron specific genes, preventing their expression^10^. REST, is highly expressed in non-neural cells as well as stem cells, while exhibiting quite low expression in neurons and other neural cells^11^. REST acts as a tumor suppressor in several carcinomas of the lung, breast and prostate^12^. Expression of an abnormal truncated splicing variant of REST (sREST) has been reported in SCLC^13^. Since sREST competes with the binding of REST to its target genes it permits abnormal gene expression through de-repression, resulting in a loss of tumor repressor expression^14^. Cell differentiation of SCLC is mediated by REST^15,16^. Suppression of REST function causes SCLC neuroendocrine phenotypes to appear. Thus, the regulation of the REST system acts as an important modulator of gene expression and phenotypes in SCLC. Misregulation of alternative splicing occurs in SCLC as well as in several other tumor types^17^, and represents a key to the progression of tumors to an aggressive phenotype. However a key factor regulating alternative splicing of REST had not been identified.

The novel splicing activator SRRM4, which was cloned from brain, activates the splicing of REST to its truncated isoform sREST^18^. Various neuronal marker genes highly expressed in SCLC correlate with the expression of sREST^15,16^. Importantly, SRRM4 induces SCLC tumor formation^19^. Expression of SRRM4 and the loss of REST activity promotes the emergence of the neuroendocrine phenotype in SCLC. Therefore, SRRM4 appears to be a novel therapeutic target for treating SCLC and preventing it to develop into an aggressive form.

To target SRRM4 specifically in the nucleus, we selected antisense technology. The use of antisense oligonucleotides (ASOs) for clinical applications is becoming a reality^20^. ASOs against various disorders are in different phases of clinical trials^21,22^, and some nucleic acid medicines have been approved for the treatment of human disorders^23^. An ASO is a single-strand nucleic acid that binds specifically to target mRNA sequences inhibiting expression of the target gene by inducing mRNA degradation by RNase H^24^. SCLC expresses abnormally higher RNaseH, suggesting application of an ASO might be a good strategy. However, no effective ASO for cancer has yet to be approved. Unmodified ASOs show limited effectiveness *in vivo* due to their vulnerability to endogenous nucleases^25^. Various studies report the development of effective ASOs, one of which is a gapmer structure^26^. As a way to avoid nuclease sensitivity the gapmer ASO (gASO) have been developed that contain a central block of DNA with a wing region of artificial nucleotides exhibiting high affinity for its target mRNA.

We have employed locked nucleic acids (LNAs)^27,28^ and artificial amido-bridged nucleic acids (AmNAs)^29^ for gASO synthesis with a phosphorothioate-linked structure that results in high affinity and nuclease resistance with low toxicity. The studies reported here represent an initial step in developing an anti-cancer ASO, in this case for SCLC. In the course of our studies we found a miRNA specifically detected in SCLC patients that can serve as a diagnostic marker for SCLC.

Here we report that the administration of SRRM4-targeted gASOs coupled with our early diagnostic methodology represents a potential breakthrough in the identification and treatment of SCLC.

## Results

### Splicing activator SRRM4 is induced in SCLC

**Figure 1:**
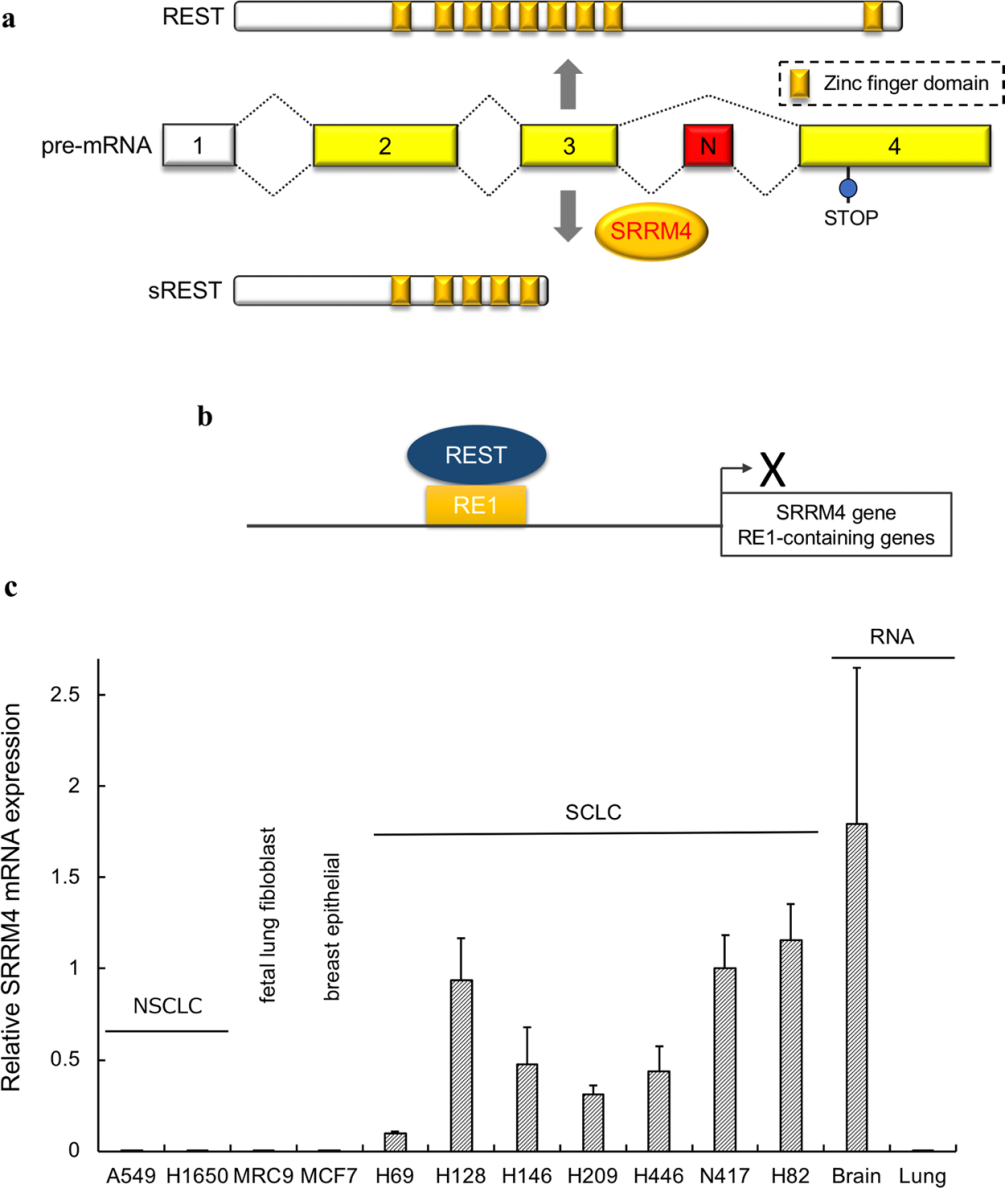
SRRM4 gene expression analysis in a spectrum of tumour cell lines. (**a**) Splicing model of REST is shown. A REST isoform (sREST) is produced by SRRM4 incorporating the N exon between exon 3 and exon 4. (**b**) Schematic of REST silencing RE1-containing genes such as SRRM4. (**c**) NSCLC cell lines (H1650 and A549), SCLC cell lines (H69, H128, H146, H209, H446, N417 and H82), fetal lung fibroblast (MRC9) cells and breast epithelial cells (MCF7) were analyzed. Total RNA from normal human brain and lung was also studied. Quantitative RT-PCR was performed and 2^−ΔΔct^ values of SRRM4 mRNA expression normalized with endogenous actin in SCLC standardized to the value of N417 as 1.0. Data are mean ± S.D. (*n* = 3).

The REST gene is normally transcribed without its N-exon, however the splicing activator SRRM4 produces sREST by facilitating alternative splicing using the N-exon (Fig. 1a). REST suppresses the expression of SRRM4 by binding to the RE1 element in its gene, thereby indirectly preventing expression of sREST^18^ (Fig. 1b). However, high SRRM4 expression in SCLC overcomes this suppression leading to sREST expression which is increased in SCLC^13^. By blocking the action of REST, sREST leads to neuronal gene expression and thus the neuroendocrine-like properties of SCLC. It is suggested that SRRM4 might be abnormally expressed in other aggressive types of neuroendocrine tumors, which are chemotherapy resistant tumors^30,31^.

After SRRM4 was first cloned from brain, we reported that SRRM4 expression was abnormally induced in SCLC and caused tumorigenesis^19^. Expression of SRRM4 in SCLC cells is coincidence with sREST expression^13,32^. We thus analyzed SRRM4 mRNA expression in several SCLC cell lines as well as in NSCLC cell lines and compared levels normalized to total RNA from normal brain and lung (Fig. 1c). Expression of SRRM4 was found to be high in human brain while almost absent in normal lung. SRRM4 expression was found to be induced in SCLC cell lines, but not in NSCLC cell lines, correlating with high sREST expression^19^. Similarly, SRRM4 mRNA is barely detectable in breast epithelial cells (MCF7) and lung fibroblast cells (MRC9). Gene expression analysis of 81 human SCLC primary tumours with RNA-seq data showed that abnormal high expression of SRRM4 corresponds to lower REST expression^6^.

### Knockdown of SRRM4 by gASOs induces cell death of SCLC

**Figure 2:**
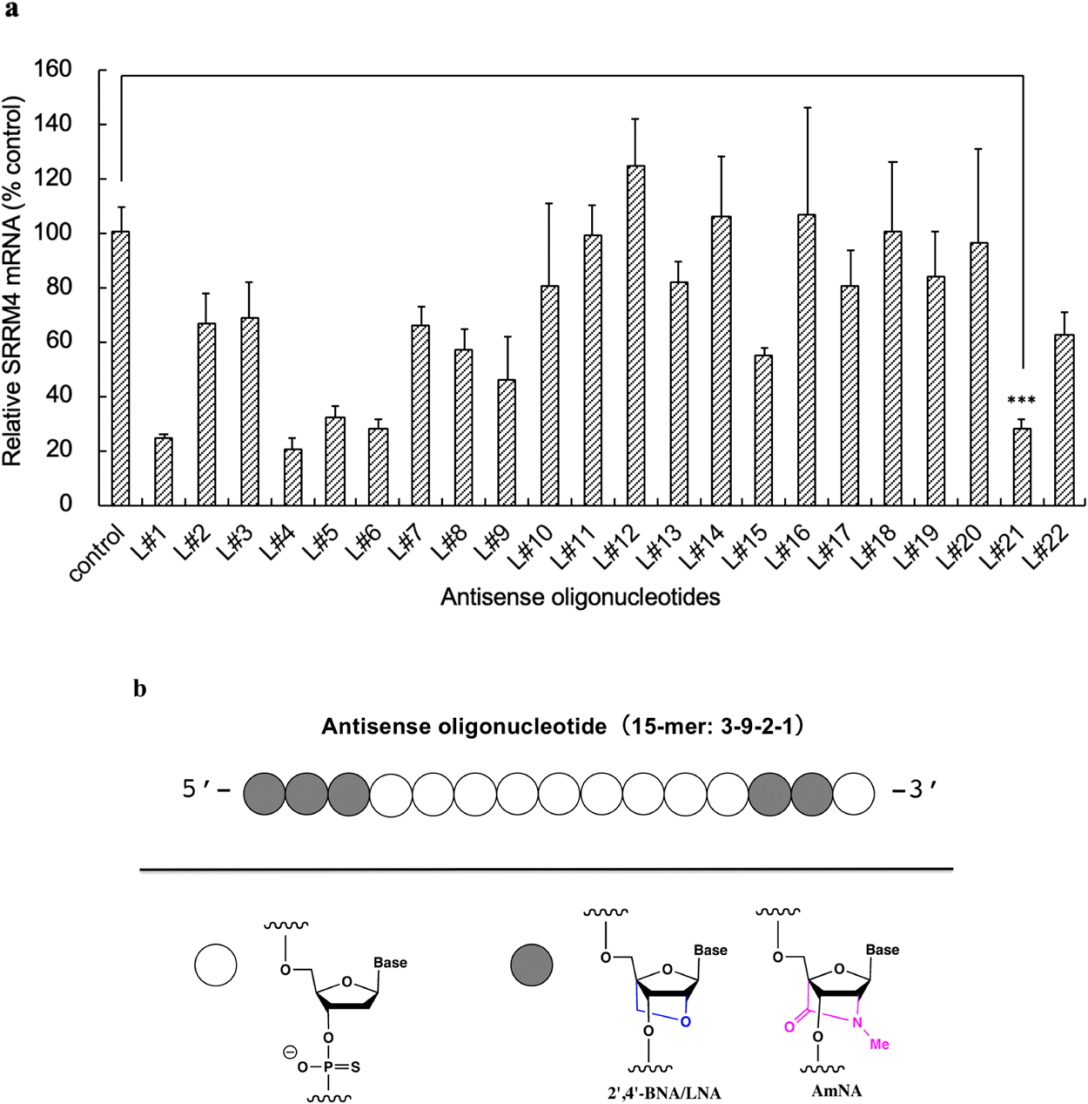
Analysis of SRRM4 mRNA in SCLC cells after transfecting ASOs. (**a**) Analysis of SRRM4 mRNA expression in N417 cells. N417 cells were transfected with antisense nucleotides (ASOs) (L#1-L#22) and a non-specific control oligonucleotide. Cells were cultured on a plastic dish for 48 hours after transfection and total RNA was prepared. qRT-PCR was conducted using specific primers. The value of qRT-PCR is shown as the relative expression using as a reference (value of 100) data from N417 cells cultured as suspension cells on a plastic plate. (**b**) Schematic of gASO. Example of the gASO contains 15 oligonucleotides with 3 artificial nucleic acids (ANA) followed by 9 natural nucleic acids (NNA), 2 ANAs and 1 NNA. We used LNA or AmNA as an ANA. Initial screening used LNA as ANA. Data are mean ± S.D. (*n* = 4). Statistic significance on L#21 which was further studied was shown. ***P < 0.001 (*t* test).

We further found that an siRNA targeting SRRM4 mRNA *in vitro* reduces the expression of SRRM4 and induced cell death in targeted cells (Extended Data Figs 1 and 2). Due to the instability of siRNAs *in vivo* we designed gapmer antisense oligonucleotides (gASOs) targeting SRRM4 mRNA. To produce effective gASO sequences we selected 15-mers of ASO candidate sequences eliminating potential toxic sequences such as specific repeat nucleotides and putative higher order RNA structures. We transfected SCLC cells with each gASO and analyzed SRRM4 mRNA by qRT-PCR (Fig. 2a) for its effectiveness in reducing SRRM4 mRNA. We evaluated the effectiveness by *in vitro* analysis of several cell lines using different culture protocols such as floating versus attached cells (Extended Data Fig. 3). For the initial screening, we used 15-mer gASOs containing artificial nucleic acids LNA at both ends (Fig. 2b). Then the length of the gASO was optimized using LNA and AmNA as artificial nucleic acids (Extended Data Figs 4 and 5). AmNA is an amido-bridged nucleic acid, which shows higher duplex stability and higher nuclease resistance^29^. Amongst the various gASO sequences that decreased SRRM4 expression, the most stably effective ones were selected for further study along with a non-specific control sequence (L26). Repression of SRRM4 by the gASOs was shown to be dose dependent (Extended Data Figs 5 and 6).

### *In vivo* antitumor effect of gASOs targeting SRRM4

**Figure 3:**
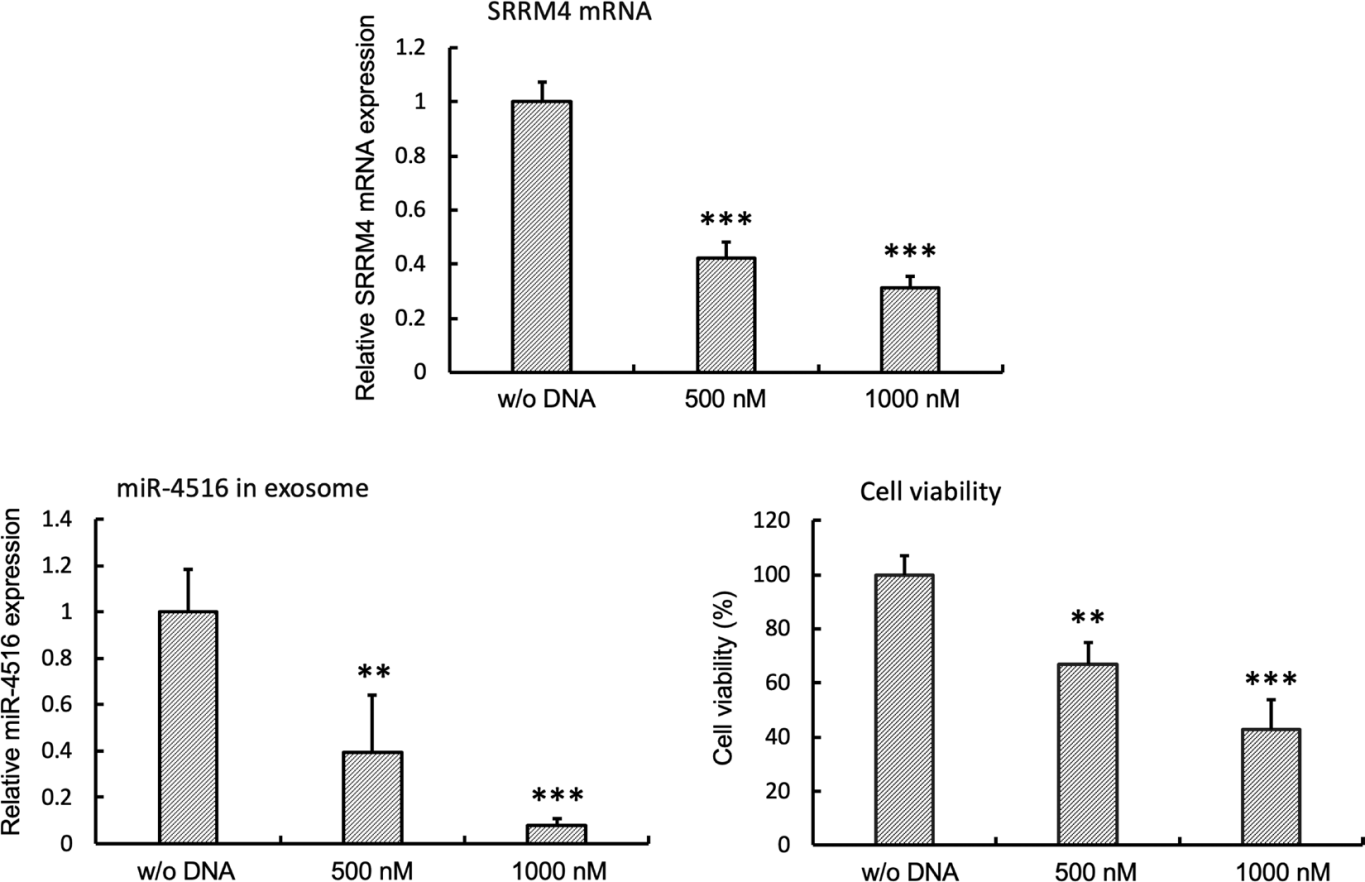
gASO inhibits cell viability and miR-4516 expression through repressing SRRM4 mRNA expression. SCLC cells (1 × 10^6^ cells/60 mm plate) were transfected with the gASO (500 or 1000 nM in 100 μl via electroporation) and cells and medium were collected after 48 hours following transfection. And then relative SRRM4 mRNA, relative miR-4516 expression and cell viability were analyzed. Data are mean ± S.D. (*n* = 3). ***P < 0.001; **P < 0.01 (*t* test).

**Figure 4:**
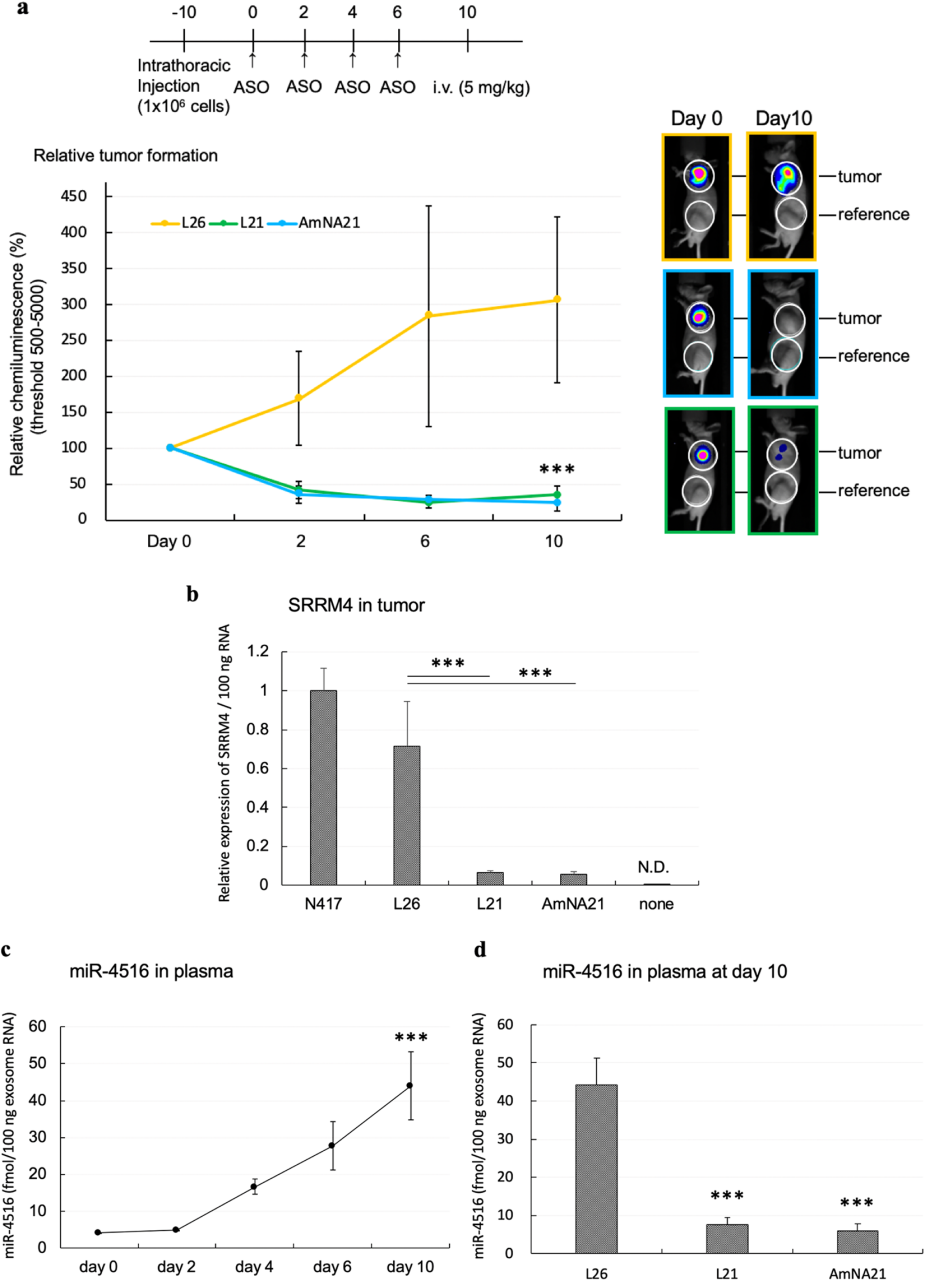
*In vivo* imaging of SCLC tumor-bearing nude mice. (**a**) Intrathoracic transplantation of SCLC cells containing the luciferase gene was performed. After 10 days of transplantation, gASO (L21) (5 mg/kg) was administered via intravenous injection every 2 days, total 4 times. *In vivo* imaging was performed at day 0, 2, 6 and 10 comparing signals of tumors after subtracting the control signal. Images at day 0 or day 10 are shown on the right. Data are mean ± S.D. (*n* = 4). (**b**) Tumor sections in the circle (see above) were analyzed for expression SRRM4 by qRT-PCR. Data are mean ± S.D. (*n* = 4). (**c**) The level of miR-4516 at day 0, 2, 4, 6 and 10. Data are mean ± S.D. (*n* = 3). (**d**) miR-4516 in plasma analyzed at day 10 after administration of gASOs. Data are mean ± S.D. (*n* = 4). ***P < 0.001 (*t* test).

*In vitro* analysis showed a gASO targeting SRRM4 mRNA successfully reduced SRRM4 expression followed by cell death (Fig. 3, Extended Data Fig. 7). Pathway analysis of SCLC cells transfected with our gASOs revealed that several apoptosis pathways were activated (Extended Data Fig. 8). In our previous study, SCLC cells expressing high levels of SRRM4 were implanted subcutaneously and were found to induce tumor formation^19^. Here we constructed human SCLC cells expressing luciferase (hSCLC-LUC) for *in vivo* imaging. We analyzed *in vivo* tumor suppression by gASO with a mouse xenograft model. After transplanting hSCLC-LUC cells subcutaneously, gASOs (L1, L4, L21 as well as a control L26) were injected intraperitoneally. After gASO administration, the chemiluminescence signal due to the expressed hSCLC-LUC was significantly decreased relative to a control gASO (L26) (Extended Data Fig. 9). Next, orthotopic implantation using intrathoracic transplantation was performed^33^. After 3 days of tumor transplantation, gASO was administered to the respiratory tract by a microinjector on subsequent days and *in vivo* imaging was performed at day 10. Administration of gASO L21 blocked or reduced tumor growth significantly, while the tumor significantly increased with the control gASO L26 (Extended Data Fig. 10). Furthermore intravenous administration of gASOs instead of administration intraperitoneally also effectively reduced the tumor signals (Fig. 4a). After 10 days of treatment with gASO, tumors were analyzed for SRRM4 expression by qRT-PCR (Fig. 4b). In the tumor part, SRRM4 mRNA was decreased or not detected after gASO administration. Although described in detail below, we found the SCLC-specific miRNA miR-4516 increased in plasma with progressing tumor formation during the time course of study (Fig. 4c). *In vivo* analysis of the gASO treated mice showed that the marker miR-4516 concurrently decreased in plasma, while control gASO (L26) did not reduce miR-4516 in plasma, instead it significantly increased (Fig. 4d).

### SCLC-specific miRNAs in patient serum as novel therapeutic biomarker

For *in vitro* studies, SCLC are cultured as floating cells while they can also be cultured as attached cells on Matrigel-coated dishes. Matrigel, extracellular matrix solubilized basement membrane preparations extracted from the Engelbreth-Holm-Swarm mouse sarcoma promoted tumor progression and the expression of SRRM4^19^. Culturing SCLC on Matrigel induced the expression of SRRM4 and correspondingly sREST as well as other RE1-controlled genes (Extended Data Fig. 11). Within SCLC cells the degree of SRRM4 expression varied suggesting that it may contribute to the heterogeneity of tumor progression and toxicity in patients. Microarray analysis showed specific miRNAs decreased in N417 cells grown with Matrigel compared with N417 cells grown without Matrigel as floating cells. The decreased miRNAs in N417 cells cultured on Matrigel are shown in Extended Data Fig. 12. We chose specific miRNAs that decreased in SCLC cells with Matrigel culture that correlated with induced SRRM4 expression. Thus expression of miRNAs, which may control SRRM4 mRNA expression were identified by microRNA target prediction coupled with a functional database (mirdb.org). We then analyzed four of these miRNAs (miR-4279, -4419b, -4516, -4635) for their secretion into the culture medium of SCLC cells with or without Matrigel. We found the miRNAs in medium accumulated at higher levels in Matrigel cultured cells at days 3 and 5 (Extended Data Fig. 13a). Most miRNAs were incorporated in exosomes (ex) while free miRNAs found in the media (sup) were thought to be due to breakage of exosomes during their isolation (Extended Data Fig. 13b). Introducing the gASO decreased SRRM4 expression as well as viability dose-dependently and the amount of miR-4516 in exosomes (Fig. 3). Extracellular matrix proteins such as Matrigel protects SCLC cells against cell death, suggesting that specific miRNA expression might cause SCLC drug resistance and cancer growth^34^. Secreted exosomes can be incorporated into a recipient cell^35^, further suggesting that the expression of these miRNAs might be important in controlling disease state.

**Figure 5:**
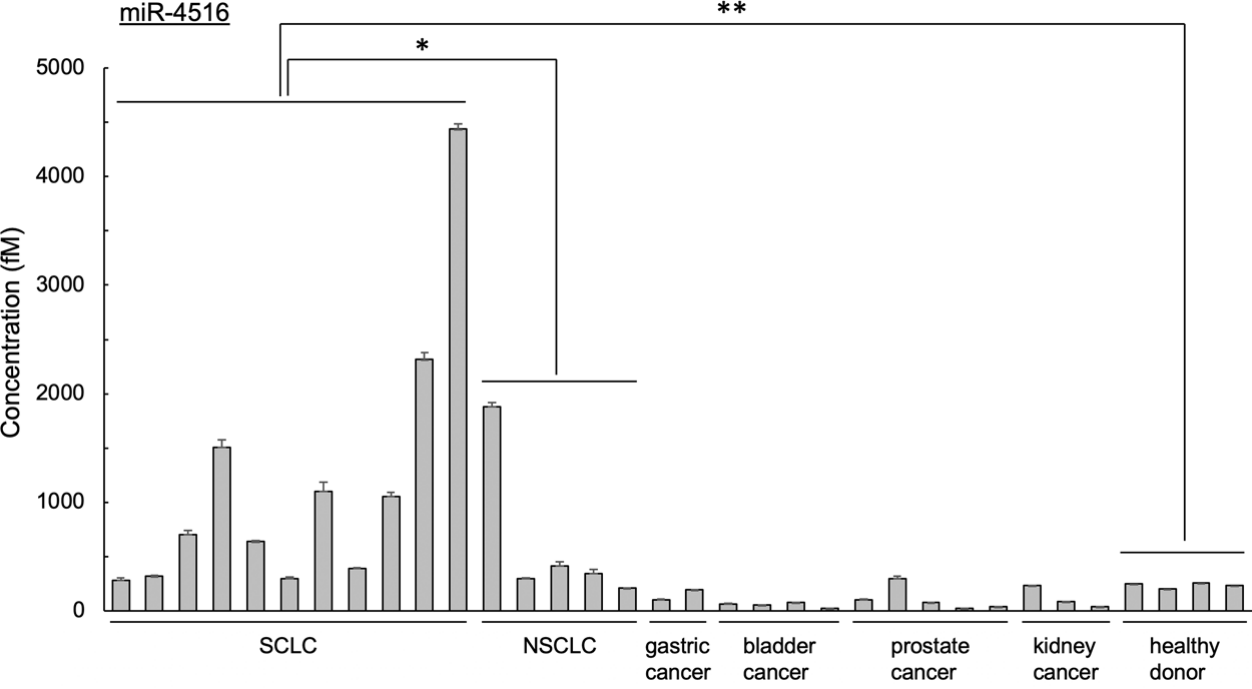
Analysis of miRNAs incorporated in exosome from cancer patient serum. Micro RNA-4516 in serum from various patients was quantitated as described in Materials and Methods. miR-4516 levels in patients with various tumors (SCLC, NSCLC, gastric cancer, bladder cancer, prostate cancer, kidney cancer) and healthy donors. The level of miR-4516 was analyzed using specific primers as described. Data are mean ± S.D. (*n* = 3). **P < 0.01; *P < 0.05 (*t* test).

Four of the miRNAs were analyzed in the serum of patients. We detected high levels of miR-4279, miR-4419b, miR-4516, and miR-4635 in serum exosomes in SCLC patients compared to other tumors. Among these four miRNAs miR-4516 showed the highest expression and was further analyzed in the serum of other patients with tumors (Fig. 5, Extended Data Fig. 14). The level of miR-4516 is significantly higher in SCLC patient serum than in other tumor bearing patient’s serum as well as in the serum of healthy persons. Some NSCLC patients show slightly higher miR-4516 expression than others suggesting that these patients may harbor more aggressive SCLC tumors. However, due to the small numbers of samples, we have not been able to establish as correlation between miR-4516 and disease severity. Lung cancer tumors normally including SCLC and NSCLC tumors show heterogeneity, complicating clinical treatment. As tumors progress, SCLC and NSCLC might be transformed to the more aggressive neuroendocrine type, in which the key switching molecule is REST^36^.

miR-4516 causes autophagy^37^, suggesting that miR-4516 decreases REST expression and subsequently induces SRRM4 expression. As noted REST represses SRRM4 through its RE1 element and represses most neuronal genes as well (Fig. 1b). The observed aberrant autophagy reportedly is a key factor in the transition to an aggressive type of tumor^38^. Interestingly, in aggressive breast cancer the expression of REST is diminished and the REST isoform sREST is detected^30^ indicating that the transition to an aggressive form in breast cancer may follow a similar mechanism as in SCLC. Furthermore, REST activity is also decreased in prostate cancer^12^ suggesting similar mechanisms to transitioning to aggressive cancer forms.

SRRM4 was detected in late castration resistant prostate cancer (CRPC)^31^ and miR-4516 was reported as a biomarker in pulmonary fibrosis^39^. High expression of miR-4516 was associated with the in-filtrative growth of the follicular variant of papillary thyroid carcinoma^40^. Recently, miR-4516 was shown to target and reduce the expression of junctional proteins such as nectin-1, claudin-4, and E-cadherin^41^, suggesting expression of miR-4516 facilitates rapid invasion and metastasis of SCLC, forming an aggressive type of tumor. Thus, analysis of miR-4516 appears to be a novel therapeutic diagnostic method, and warrants analyzing many more patients including late breast cancer and CRPC patients. Interestingly significant levels of miR-4516 was detected in the blood of a healthy person living in a town with a high level of pollution by air particles with a diameter of less than 2.5 micrometers (PM2.5)^37^, which as noted is a particle accumulating in the respiratory tract and causing lung cancer. The number of children recently diagnosed with lung cancer is increasing^42^, proposed to be caused particularly by PM2.5. Most lung cancers are associated with inhalation of tobacco smoke, but individuals who have never smoked also develop lung cancer possibly due to air pollutants^43^. Thus, PM2.5 has been categorized as a high-risk factor for developing lung cancer by the World Health Organization^44^. It will be interesting to follow this and similar patients to see if they develop an aggressive form of SCLC.

As described above, gASO against SRRM4 successfully reduced the tumor formation *in vivo* by suppressing specifically SRRM4 expression. SRRM4 is abnormally expressed in SCLC but not in most of normal tissues other than brain. As naked gASO does not go through blood-brain barrier, gASO will be useful for clinical treatment. We are currently studying on the pharmacokinetics and toxicity of gASO for future clinical application.

### Implications

This work demonstrates that antisense DNA technology is potentially applicable to the treatment of SCLC. Administration of gapmer ASOs have the potential to reduce SCLC tumor formation and a SCLC-specific miRNA in patients. In our model system a gapmer ASO reduced both an miRNA in plasma and tumor formation suggesting both a new treatment paradigm as well as a new diagnostic tool. We anticipate that this work will contribute to the future development of both treatment paradigms and diagnostic tools for SCLC.

## Materials and Methods

### Cell culture

All cell lines were purchased from the American Type Culture Collection (ATCC), JCRB cell bank and LONZA. All SCLC cell lines were cultured as floating aggregates grown in RPMI-1640 medium containing 10% FBS, L-glutamine (2 mmol/L), penicillin (100 U/ml) and streptomycin (100 μg/ml). Cells were incubated in a humidified atmosphere with 5% CO_2_. All cells were cultured according to the manufacture’s instruction. The cell lines used are the following; SCLC cell lines: H69 (HTB-119), H128 (HTB-120), H146 (HTB-173), H209 (HTB-172), H446 (HTB-171), N417 (CRL-5809), H82 (HTB-175), STC1 (JCRB1053); NSCLC cell lines: A549 (CCL-185), H1650 (CRL-5883); Fetal lung: MRC9 (CCL-212); Breast cancer: MCF7 (HTB-22); NHLF (CC-2512); NHBE (CC-2540).

### *In vitro* transfection of ASOs

For transfecting floating SCLC cells, cells (1 × 10^6^ cells) were transfected using Nucleofector according to the manufacture’s instruction (LONZA) and cultured in 2 ml of culture medium. For attached cells, transfection using Lipofectamine 3000 was performed according to the manufacture’s instruction (Thermo Fischer Scientific). Briefly, cells confluence of 70–80% in a culture dish were transfected as described in manufacture’s instruction. Transfection of ASOs into STC1 and H82 was also performed using Ca^2+^ enrichment of medium (CEM) method.

### Human serum

Human blood donations and all the donors were asked to sign a written consent approved by the Medical Ethics Committee of Kansai Medical University (#1325). All donors provided their informed consent in writing. Serum donated from healthy individuals were obtained base on “Guidelines on the use of blood donation etc for research and development” from the Japanese Red Cross Society. All patients’ sera were obtained at the time of first diagnosis before cancer clinical treatment. Samples from SCLC, NSCLC, gastric cancer, bladder cancer, prostate cancer, kidney cancer and healthy condition individuals were analyzed. The patient information of SCLC and NSCLC is shown (Extended Data Fig. 15). Performed experiments were approved by the Medical Ethics Committee of Kansai Medical University (#1325).

### Quantitative PCR with reverse transcription (qRT-PCR)

Total RNA was prepared according to the miRNeasy and total RNeasy kit as per the manufacturer’s instructions (Qiagen). Total RNA was quantified spectrophotometrically at 260 nm. Total RNA (1 μg) was transcribed at 42 °C for 60 min using SuperScript VILO kit (Thermo Fischer Scientific). Amplification was conducted using StepOnePlus™ Real-Time PCR Systems (Thermo Fisher Scientific). Forward and reverse primers (10 pmol each) were used in 20 μl reactions. Aliquots of cDNA (1/20 of RT) was analyzed by qRT-PCR. The qPCR was carried out with initial activation at 95 °C for 20 sec followed by 40 cycles of amplification (95 °C for 3 sec, 60 °C for 30 sec). Fluorescence development was assayed once each cycle of amplification as recommended by the manufacture. Relative mRNA levels were analyzed according to the 2^−ΔΔ^^Ct^ method, in which all ΔCt values are normalized to β-actin (actin). All qPCR experiments were carried out at least three times, and the mean ± SD values were calculated. Statistical significance was examined by paired *t*-test. PCR products were analyzed as single bands on agarose gels and by DNA sequence to confirm products. RNA from brain (CAT#636530) and lung (CAT#636524) were purchased from Takara Bio. The primer sequences are following: hSRRM4 forward: 5′-tgacaaagacttgacaccacc-3′; hSRRM4 reverse: 5′-acctgcgtcgcttgtgttt-3′; actin forward: 5′-ggccgtcttcccctccatcg-3′; actin reverse: 5′-ccagttggtgacgatgccgtgc-3′; GAPDH forward: 5′-gagtcaacggatttggtcgt-3′; GAPDH reverse: 5′- gacaagcttcccgttctcag -3′.

For qRT-PCR for miRNA, total RNA was transcribed using miScriptII reverse transcriptase (Qiagen). Universal primer and each primer for respective miRNA were added in the PCR reaction using miScript SYBR Green PCR kit (Qiagen). PCR was performed according to the manufacture’s instruction. The primers were used: hsa-miR-4516 (CAT#MS00037555), hsa-miR-4279 (CAT#MS00021322), hsa-miR-4419b (CAT# MS00041440), hsa-miR-4635 (CAT#MS00044688) (Qiagen).

### Assessment of cell viability and cytotoxicity

The viability of cells was measured by CCK8 (Cell Counting Kit-8, Dojindo) according to the manufacture’s protocol. After culturing with or without treatment in 96 well plate for appropriate culture time. The assay was performed more than three times to confirm reproducibility. Cytotoxicity was assayed with LDH assay kit (Dojindo). The absorbance at 492 nm was analyzed using SpectraMax microplate reader (Molecular Devices).

### Microarray analysis

Global miRNA expression of the samples was analyzed using the miRCURY LNA Array (Exiqon) according to the manufacture’s instruction performed at Takara Bio.

### NGS pathway analysis

GeneChip expression analysis (Human Genome U133 Plus2.0) was performed and the pathway analysis was performed using Kyoto Encyclopedia of Genes and Genomes pathway (KEGG) by Takara Bio.

### Western blot analysis

Protein lysate was prepared in RIPA buffer according to the manufacture’s instruction (Nacalai). Total protein (50 μg) was fractionated by SDS-PAGE on a 7% Protean TGX gel and transferred to nitrocellulose membrane using Transblot Turbo (Bio-Rad). Membranes were blocked with TBS (20 mM Tris-buffered saline, pH7.2) with 1.0% skim milk at room temperature (RT) and then reacted with appropriate primary antibody in TBS containing 0.1% Tween 20 (TBS-T) at 4 °C for overnight (−15 hr). After washing with TBS-T, a fluorescent-labeled secondary antibody (dilution of 1/15,000 or 20,000) was added and incubated for 1 hour at room temperature. Protein bands were visualized using an Odyssey fluorescence detection system (LI-COR). Antibodies (anti-SRRM4, abcam) were obtained from Santa Cruz Biotechnology, and an anti-β-actin antibody was from Wako Chem.

### Gapmer antisense oligonucleotides (gASOs)

Antisense DNA targeting SRRM4 mRNA was designed and constructed. Briefly, All 2′, 4′-BNA/LNA-based ASOs and modified with AmNA were synthesized and purified by Gene Design, Inc. (Osaka, Japan). Subsequently, we screened and optimized using effective LNA-ASO, which replaced LNA monomers at the flanked region of the ASOs with AmNA monomers to compare knockdown efficiency *in vitro*. The sequences are 5′-TGAACAAAATAATAC-3′ (L26), 5′-TTGTGTGACTGAAGC-3′ (L21) and 5′-TTGTGTGACTGAAGC-3′ (AmNA21). Nucleotides underlined are artificial nucleotides respectively.

### Animals

All procedures were performed under a protocol approved by the Animal Experimentation Committee at Osaka University. All mice discussed in this study were housed in AAALAC-accredited facilities, and the overall protocol was approved by the institutional care and use committee (#25-8-6). Environmental conditions within rooms were maintained at 21 ± 1 °C with 50% ± 20% relative humidity and ventilated at a minimum of 15 HEPA-filtered air changes per hour. Animals were kept on a 12:12 light: dark cycle and provided *ad libitum* access to water and feed. Athymic nude mice (7-week-old male BALB/c Slc-*nu/nu*) were obtained from Shimizu Laboratory Supplies Co. and allowed to acclimatized for 1 week prior to the experiments.

### *In vivo* tumor formation studies

N417-LUC cells were harvested by centrifugation at 1,000 rpm for 3 minutes. An aliquot of the cells disaggregated by gentle trituration was assessed for cell viability and cell number. Cells were suspended in serum-free RPMI-1640 medium, and Matrigel (BD biosciences) was mixed with an equal volume of cell suspension. To generate tumor xenografts in mice, (N417-LUC) cells (1.0 × 10^6^) were implanted subcutaneously in the mid-dorsal region of each 7-weeks-old male nude mouse (BALB/c Slc-nu/nu). Tumors were allowed to grow for 7–10 days and reach sizes between 5–7 mm before imaging. A final volume of 0.1 mL was immediately transplanted subcutaneously or intrathoracically into 7-week-old male BALB/c Slc-nu/nu athymic nude mice. ASOs (5.0 mg/kg) were administered via i.p. or i.v. every 2 days. For *in vivo* imaging luciferase activity was assessed in anesthetized mice (3–4% isoflurane). Groups of 3–5 mice at a time were administered D-luciferin (150 μl, PerkinElmer) via intraperitoneal administration. Imaging data were acquired using the IVIS Spectrum (PerkinElmer) or NightOwl (Berthold).

### Preparation of luciferase-harboring N417 cells

N417-LUC/GFP (human SCLC) cells were constructed by transducing with a lenti virus vector, luciferase(firefly)-2A-GFP(Bsd) to express a dual reporter containing firefly luciferase and green fluorescent protein (GFP) according to the manufacture’s instruction (GenTarget). Cells were cultured and selected in RPMI-1640 containing 5 μg/ml blasticidin S. After 3 weeks of culturing, the cells were analyzed by flowcytometry for GFP and luciferase assay for stable expression cells.

### Purification of exosome containing miRNAs

Total RNA in exosome was prepared using exoRNeasy Serum/Plasma Kit (QIAGEN). Briefly, prefiltered plasma was mixed with same volume of 2x binding buffer (XBP) and added to the exoRNeasy membrane affinity column. Attached vesicles to membrane were lysed by adding QIAzol to the spin column, and the lysate was collected by centrifugation. The miRNeasy Serum/Plasma Spike-In Control, miR-39 was added.

### Transfection of siRNA

Transfection of siRNA was conducted according to the manufacturer’s protocol. Briefly, cells in the exponential phase of growth were plated in 6-well cell culture plates at 1 × 10^5^ cells/well, grown for 24 hours, and then transfected with SMART-pool siRNAs (Thermo Fisher Scientific) as recommended by the manufacturer. Transfections of plasmids were conducted with the use of the DharmaFECT Transfection Reagent (Thermo Fisher Scientific) according to the manufacturer’s instructions. After 45 to 48 hours following transfection, cells were collected. Transfection efficiency was approximately 40% to 60% as judged by the fluorescence of GFP.

### Statistics

Statistical significance was assayed by Student’s *t* test with Excel (two-tailed unpaired or paired *t* test, depending on the experiment). *P < 0.05; **P < 0.01; ***P < 0.001. Data are represented as mean ± S.D.

## Supplementary information

Supplementary Data Set
