# Supplementary material for: A gapmer antisense oligonucleotide targeting SRRM4 is a novel therapeutic medicine for lung cancer
Source: Sci Rep. 2019 May 20;9:7618. doi: 10.1038/s41598-019-43100-1 (PMC6527545; doi:10.1038/s41598-019-43100-1)
Supplement: Supplementary file 1 — Supplementary Data Set [file 41598_2019_43100_MOESM1_ESM.pdf]

A gapmer antisense oligonucleotide targeting *SRRM4* is a novel therapeutic medicine for lung cancer.

Masahito SHIMOJO, Yuuya KASAHARA, Masaki INOUE, Shin-ichi TSUNODA,
Yoshie SHUDO, Takayasu KURATA, Satoshi OBIKA

Extended Data Figure 1

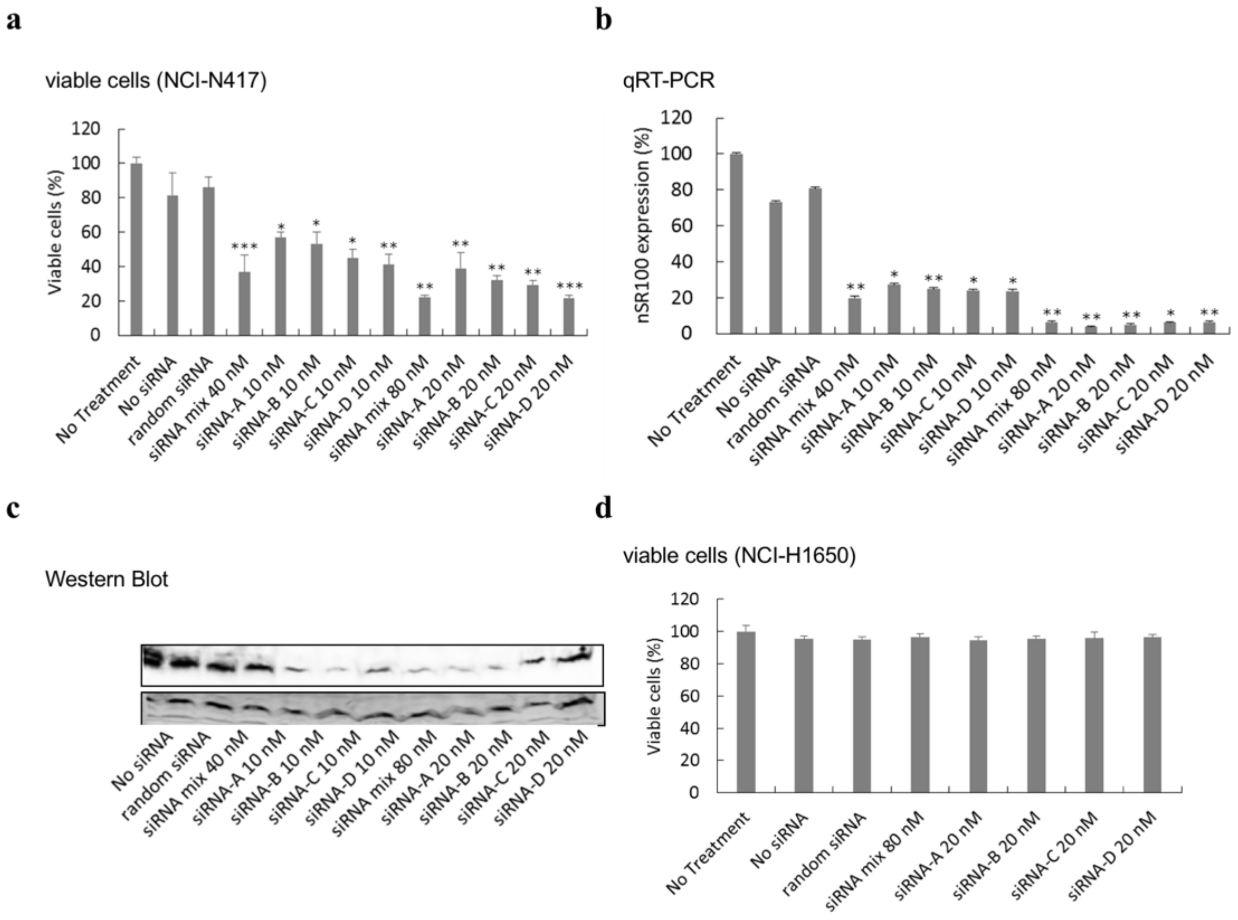

Extended Data Fig. 1. (**a**) Viability analysis of N417 transfected by SRRM4 siRNAs. N417 cells cultured on Matrigel-coated culture dishes and transfected with unrelated siRNA (scrambled siRNA) or respective SRRM4 siRNAs. After 48 hours of transfection cell proliferation was measured using the WST-8 reagent. The results of the cell proliferation assays are shown as the relative number of viable cells compared with untransfected cells, which were given a reference value of 100. ***, P<0.001; **, P<0.01; *, P<0.05 (*t* test). (**b**) Total RNA was then prepared, and RT-PCR conducted using specific primer pairs for human SRRM4. The results of qRT-PCR are shown as the relative expression level compared to actin, which was given a reference value of 1.0. Data are mean ± S.D. (*n*=3) ***, P<0.001; **, P<0.01; *, P<0.05 (*t* test). (**c**) Western blot analysis of SRRM4 in N417 cells transfected with SRRM4 siRNAs. After 48 hours of transfection with siRNA total cell lysates were prepared, and an aliquot (100 μg) subjected to SDS-PAGE, followed by Western blot analysis using anti-SRRM4 antibody and anti-GAPDH antibody as a control. (**d**) Viability analysis of H1650

transfected by SRRM4 siRNAs. H1650 cells were cultured on Matrigel-coated culture dishes for 48 hours following transfection of siRNA. Cell proliferation was measured via the WST-8 reagent. The results of cell proliferation assay are shown as the relative viable cells compared to untransfected cells, which were given a reference value of 100. Data are mean ± S.D. (n=3) ***, $P<0.001$; **, $P<0.01$; *, $P<0.05$ (t test).

Extended Data Figure 2

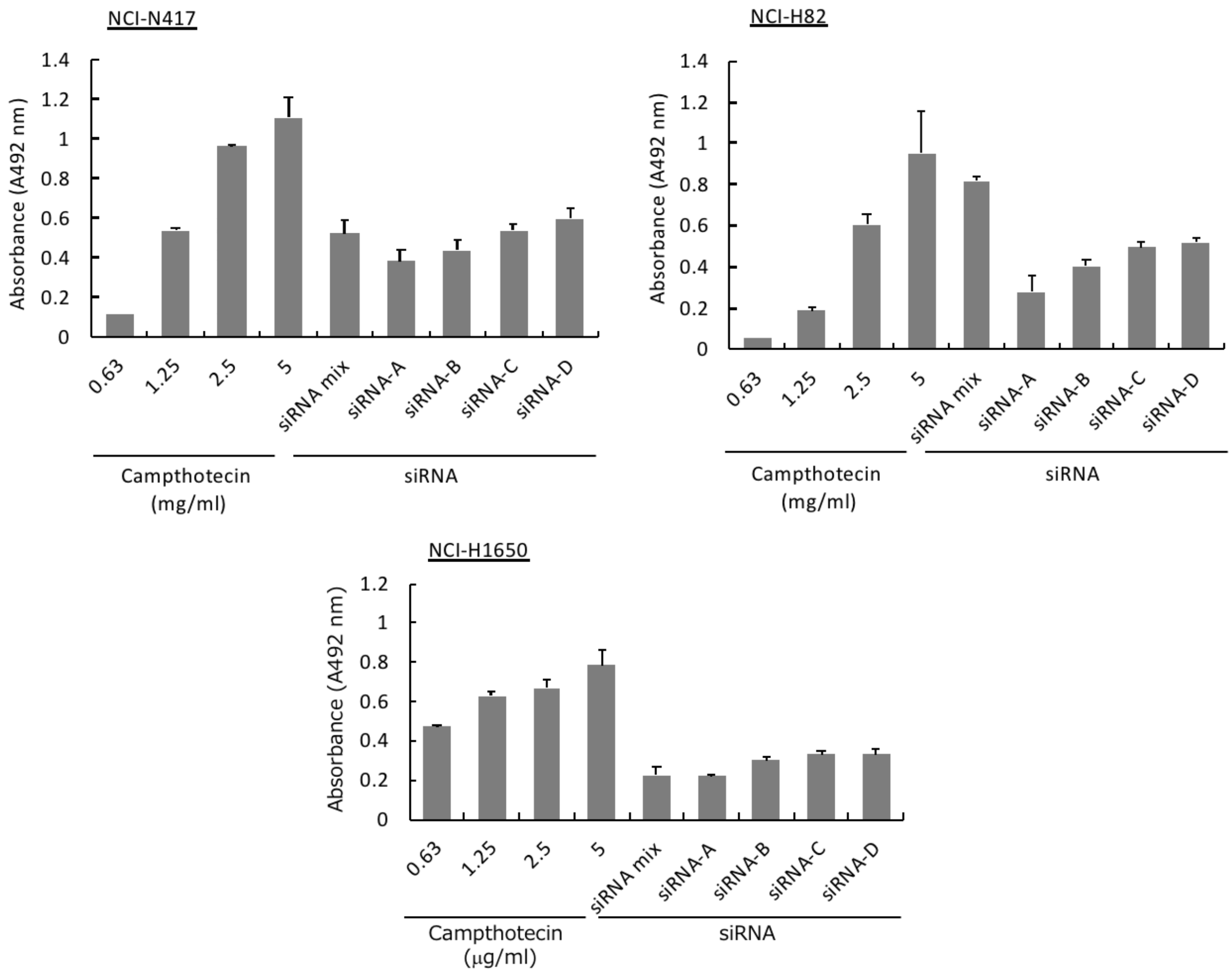

Extended Data Fig. 2. LDH activity assay of SCLC cells transfected with siRNAs for SRRM4. N417 and H82 cells were transfected with SRRM4 siRNAs and cultured for 48 hours. Cells were also cultured in medium containing camptothecin without transfection. Data are mean ± S.D. (*n*=4)

Extended Data Figure 3

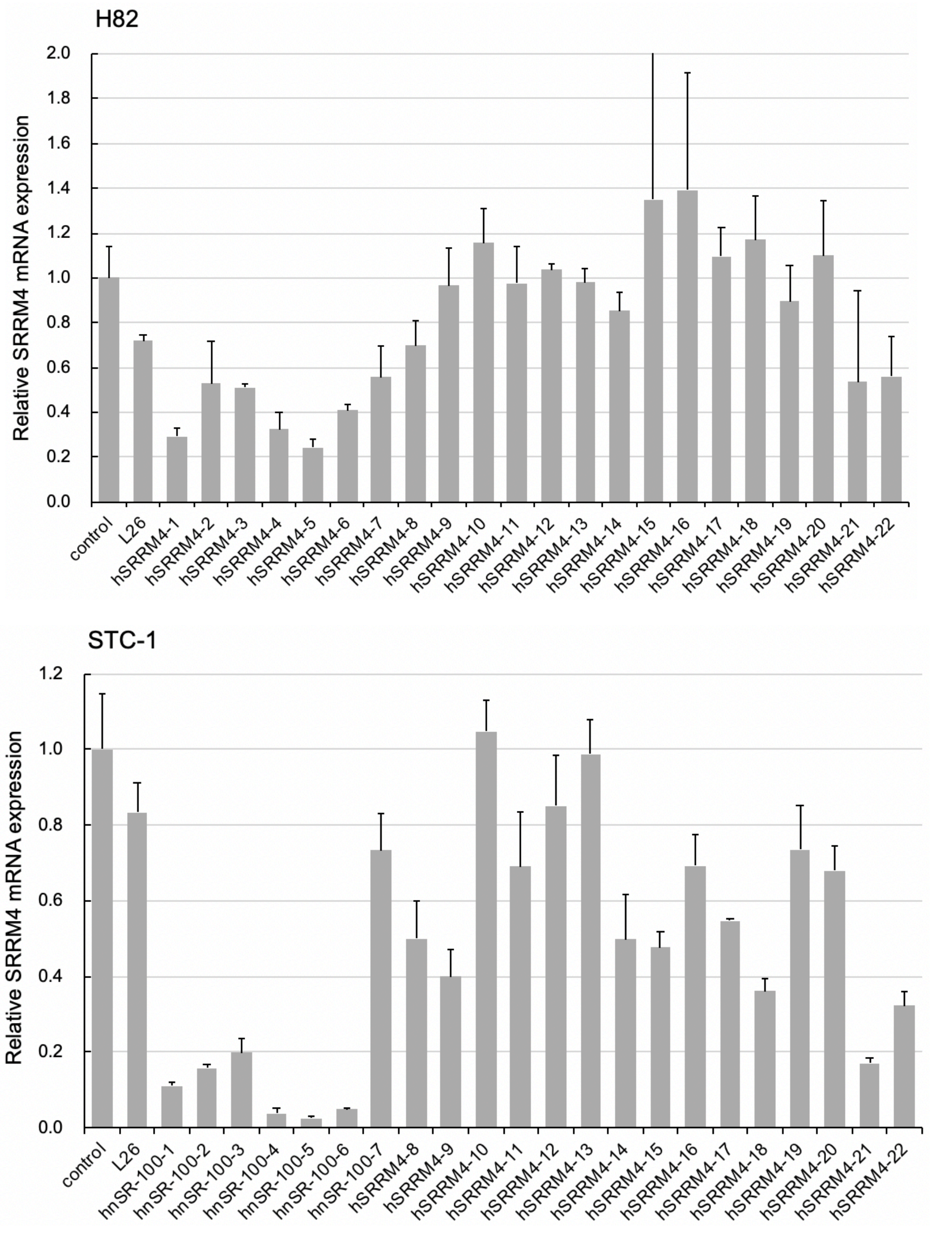

Extended Data Fig. 3. Expression analysis of SRRM4 mRNA by qRT-PCR in SCLC cells. Analysis of SRRM4 mRNA expression in H82 and STC1 cells. H82 and STC1 cells were transfected with various gASOs (L#1-L#22) and a non-specific control oligonucleotide (L26). Cells were cultured on a plastic dish for 48 hours, and total RNA prepared. The qRT-PCR was conducted using specific primers. The value of the qRT-PCR analysis is shown as the relative expression using as a reference (value of 1.0) for cells without oligonucleotide. Data are mean ± S.D. (*n*=4)

Extended Data Figure 4

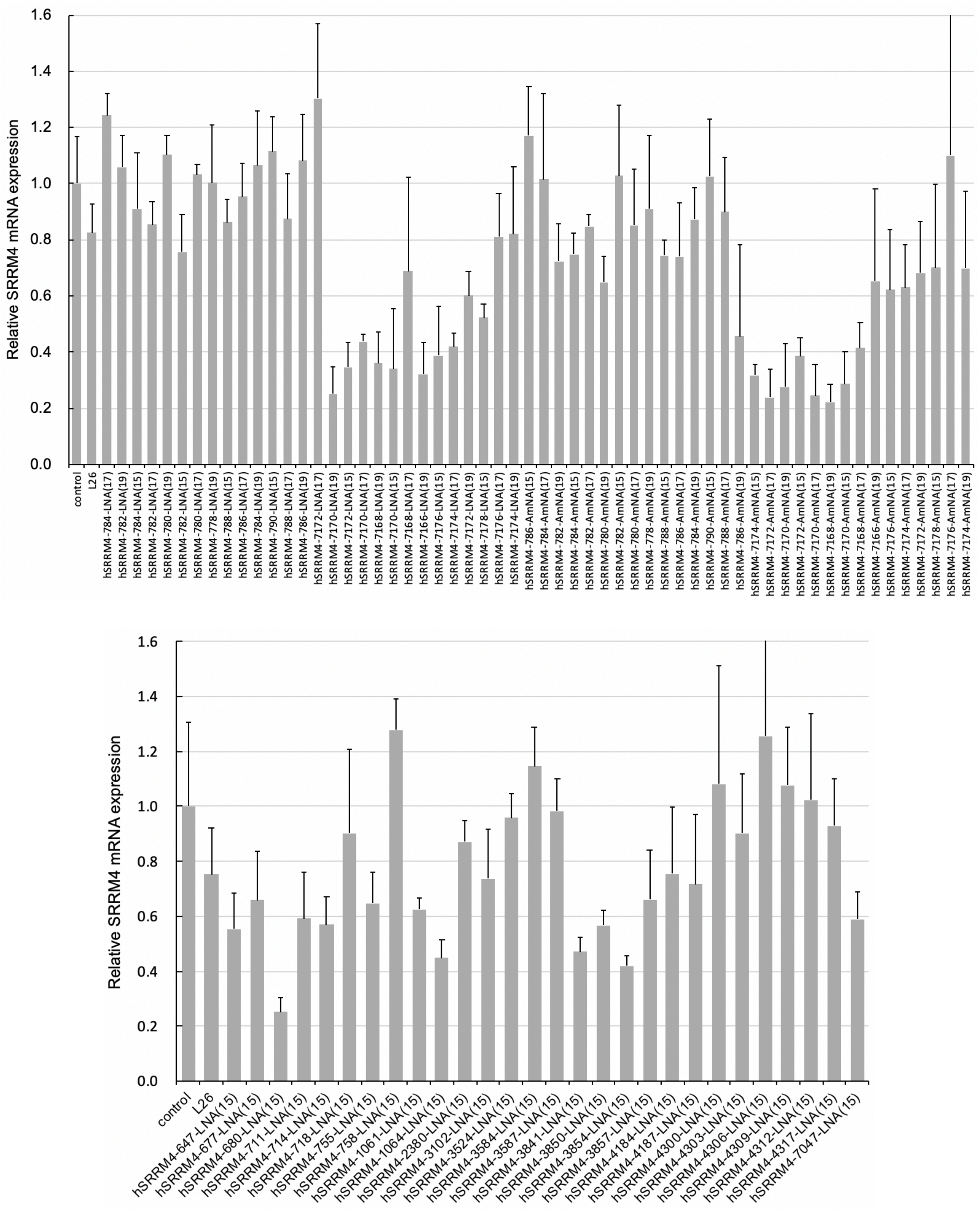

Extended Data Fig. 4. Various sequences of gASO were transfected in STC-1 and relative SRRM4 mRNA analyzed. Cells were cultured on a plastic dish for 48 hours, and total RNA prepared. qRT-PCR was conducted using specific primers. The qRT-PCR value is shown as the relative expression using as a reference (value of 1.0) data from cells without oligonucleotide. Data are mean ± S.D. (n=4)

Extended Data Figure 5

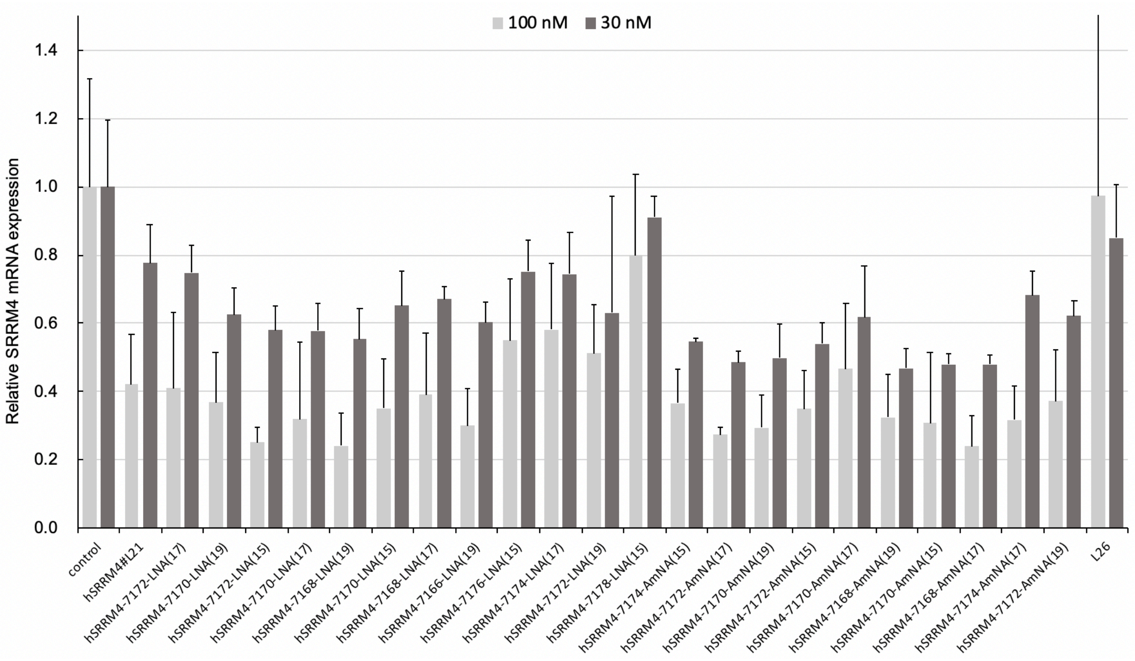

Extended Data Fig. 5. Various sequences of ASO were transfected in STC-1 and relative SRRM4 mRNA was analyzed as in Extended Data Fig. 4. Each ASO (100 or 30 nM) was used. Data are mean ± S.D. (*n*=6)

Extended Data Figure 6

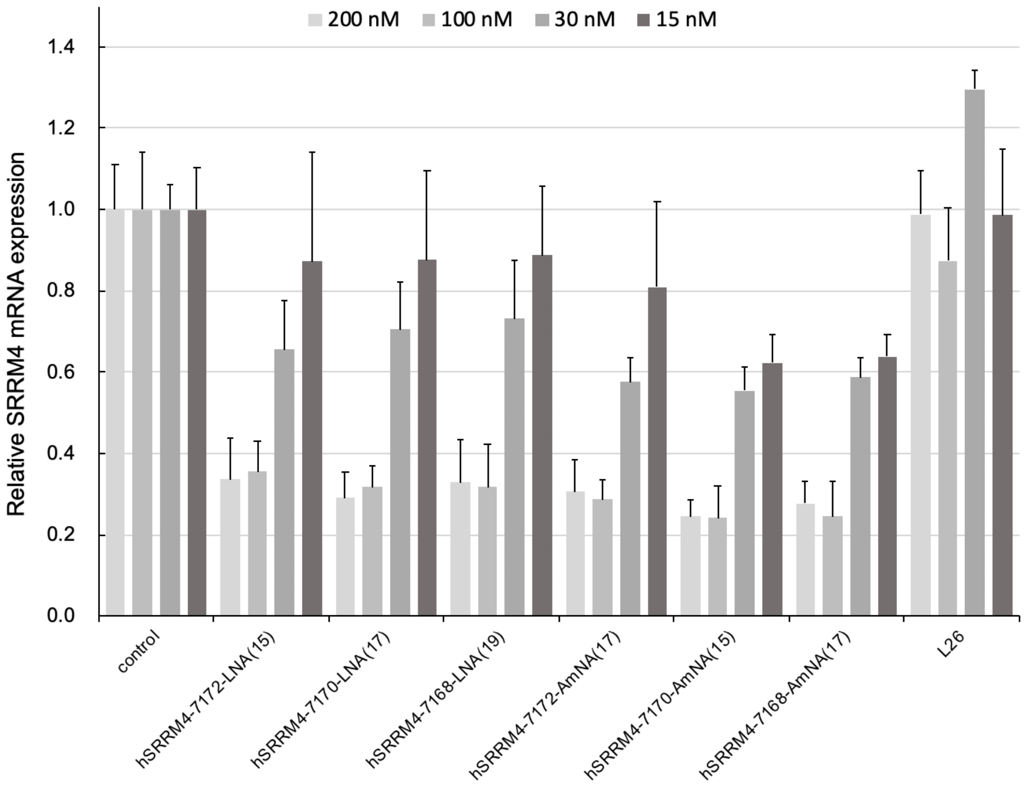

Extended Data Fig. 6. Analysis of SRRM4 mRNA in cells after transfecting gASOs. Various sequences of ASO were transfected in STC-1 and relative SRRM4 mRNA was analyzed. Each gASO was used at 200, 100, 30, or 15 nM. Data are mean ± S.D. (n=6)

Extended Data Figure 7

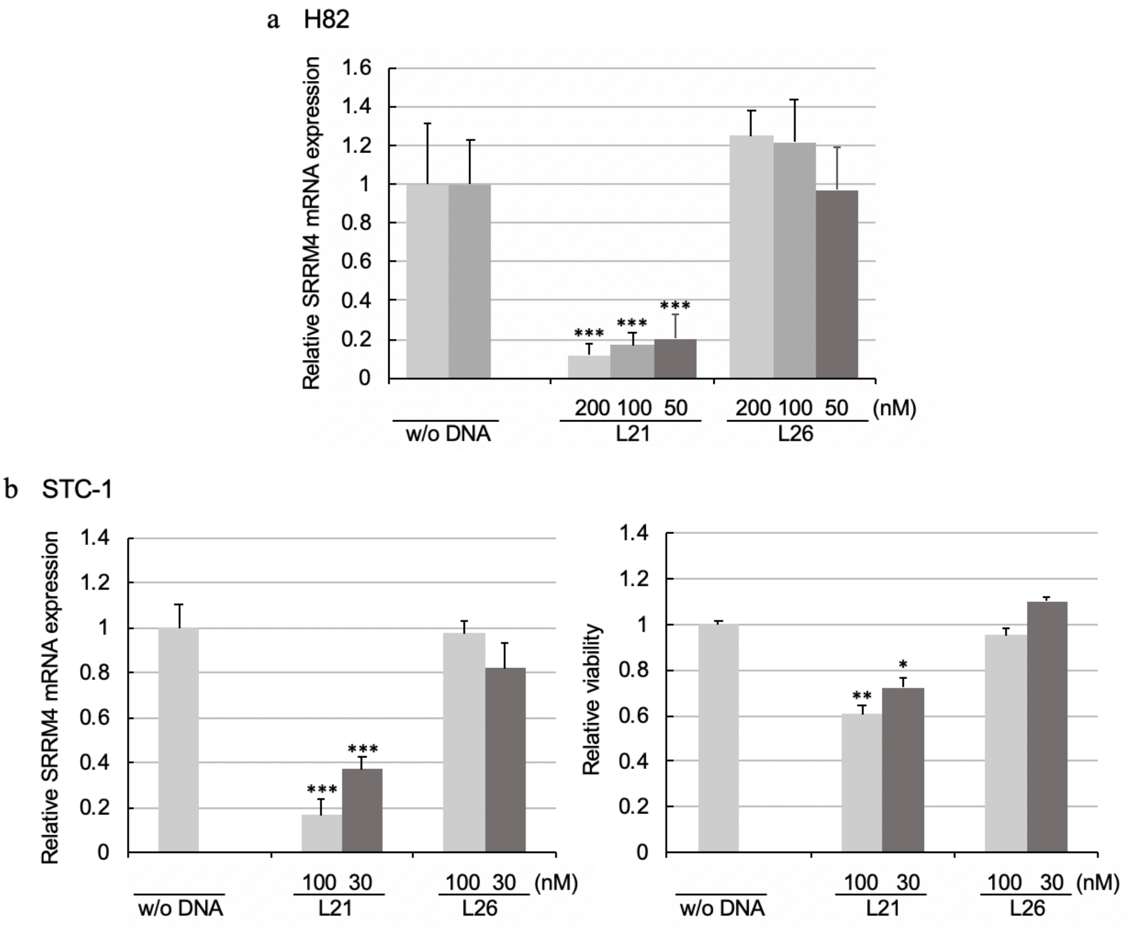

Extended Data Fig. 7. gASO inhibits SRRM4 mRNA expression and cell viability in H82 and STC-1 cells. (a) H82 cells were transfected with the gASO (200, 100 or 50 nM) and SRRM4 mRNA expression were analyzed after 48 hours of transfection. (b) STC-1 cells were transfected with the gASO (100 or 30 nM) and cell viability and SRRM4 mRNA expression were analyzed after 48 hours of transfection. Relative SRRM4 mRNA, and cell viability were shown. Data are mean ± S.D. (n=4) Statistic significance on L21 compared to each concentration of L26 was shown. ***, $P<0.001$; **, $P<0.01$; *, $P<0.05$ (t test).

Extended Data Figure 8

Pathway (Down↓) 1/2 fold

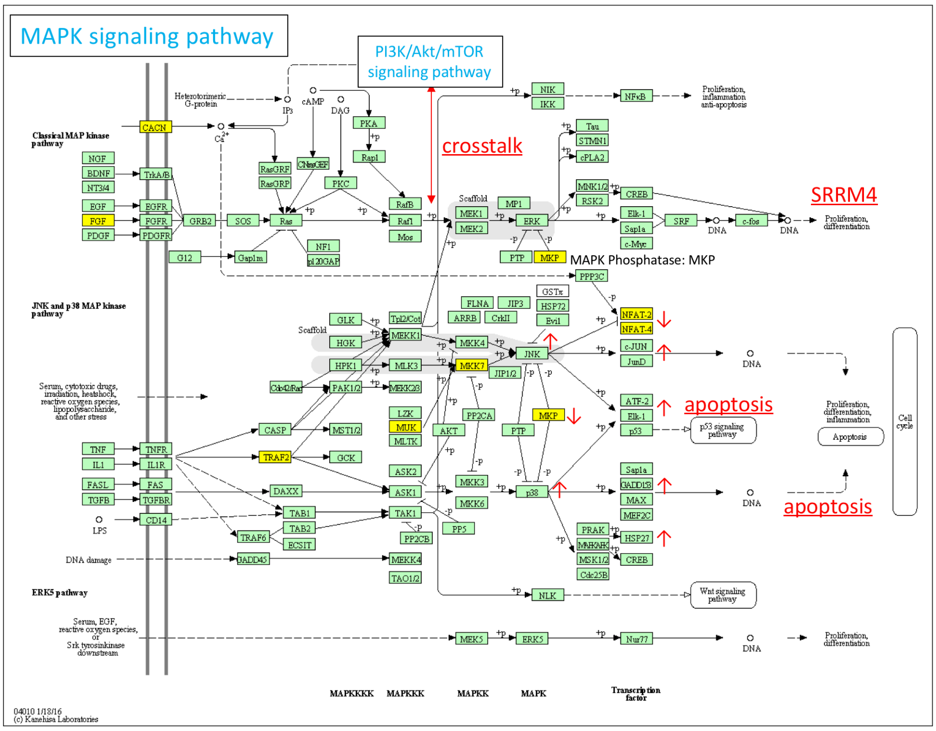

Pathway (UP↑) 2 fold

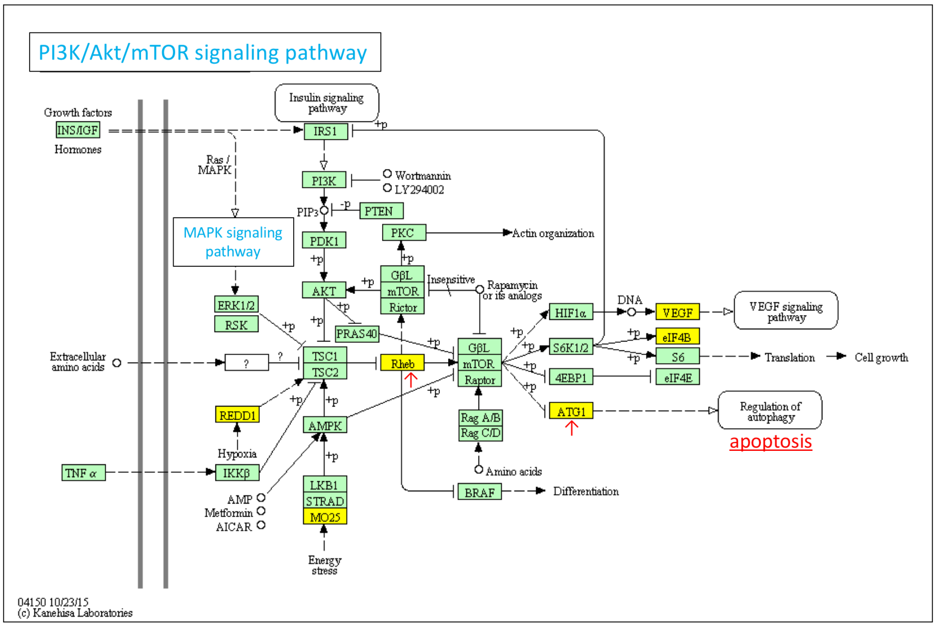

Extended Data Fig. 8. Examples of pathway analysis. N417 cells were transfected with L21 or L26 and NGS expression analyzed. Comparing control cells with L26, gene expression that increased 2 fold (↑) or decreased 1/2 fold (↓) is shown in yellow. Reference: Kanehisa, M. & Goto, S. KEGG: Kyoto Encyclopedia of Genes and Genomes. *Nucleic Acids Res.* **28**, 27-30 (2000).

Extended Data Figure 9

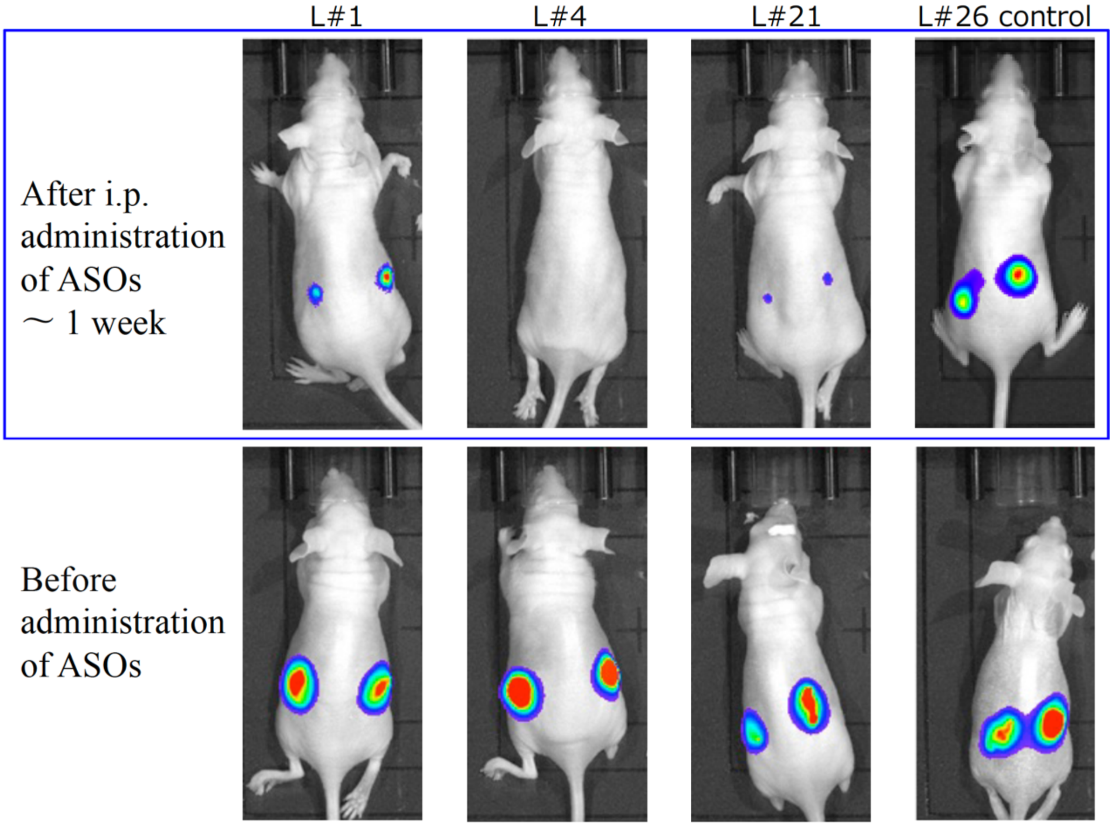

Extended Data Fig. 9. hSCLC-LUC was transplanted subcutaneously into mice and then each ASO (5 mg/kg) was administered intraperitoneally daily for 1 week. The chemiluminescence signal due to the expressed hSCLC-LUC was measured as described in Materials and Methods.

Extended Data Figure 10

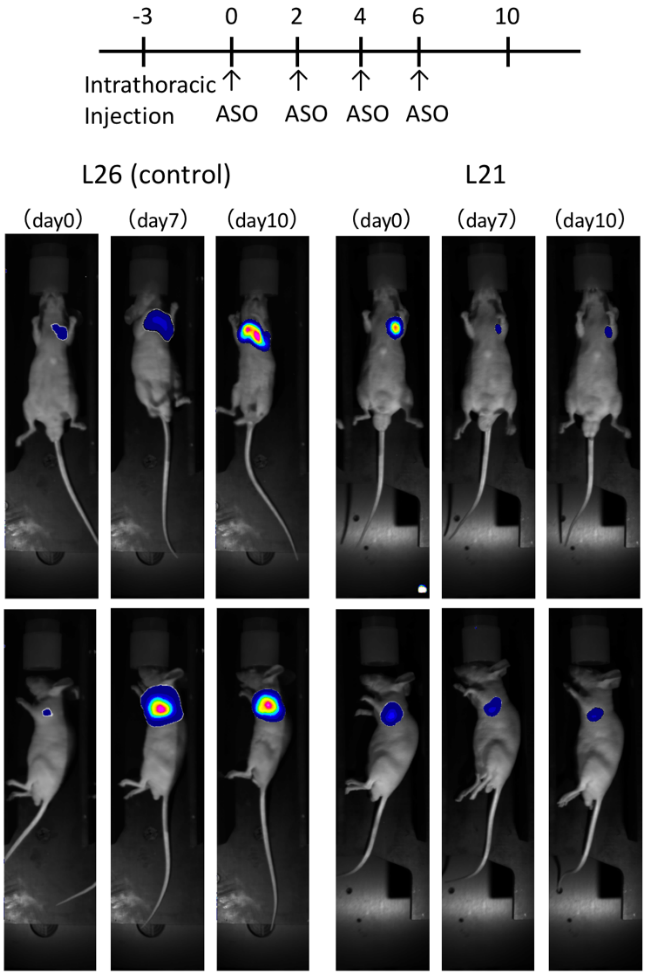

Extended Data Fig. 10. *In vivo* imaging of SCLC tumor-transplanted nude mice. Intrathoracic transplantation of SCLC cells expressing the luciferase gene was performed. After 3 days of transplantation, gASO (L21) (5 mg/kg) was administered to the respiratory tract every 2 days, total 4 times, and then *in vivo* imaging was performed.

Extended Data Figure 11

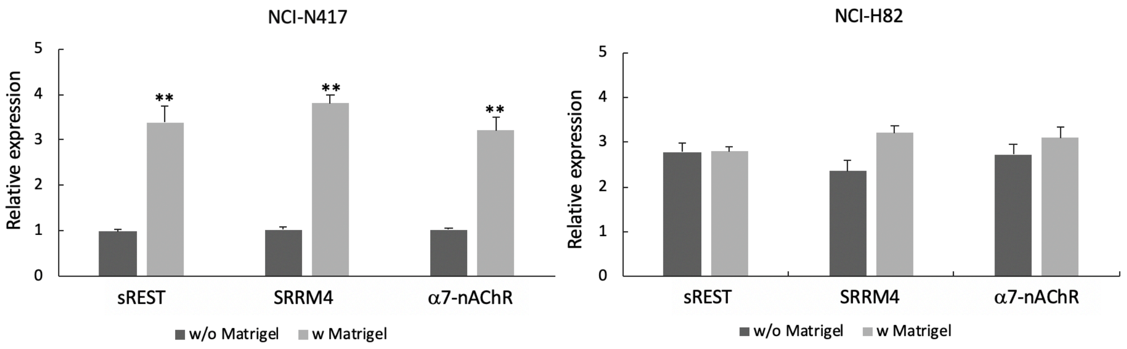

Extended Data Fig. 11. Analysis of SRRM4 expression in SCLC cells with or without Matrigel. N417 and H82 cultured with (w) or without (w/o) Matrigel on the culture dish. Expression analysis of sREST, SRRM4 and α7-nAChR by qRT-PCR was performed. Cells were cultured on a plastic dish with or without Matrigel for 48 hours, and cells were collected and total RNA was prepared. qRT-PCR was conducted using specific primers. The value of qRT-PCR is shown as the relative expression using as a reference (value of 1.0) data from N417 cells cultured as suspension cells on a plastic plate. Data are mean ± S.D. (n=4) **, $P < 0.01$ (t test).

Extended Data Figure 12

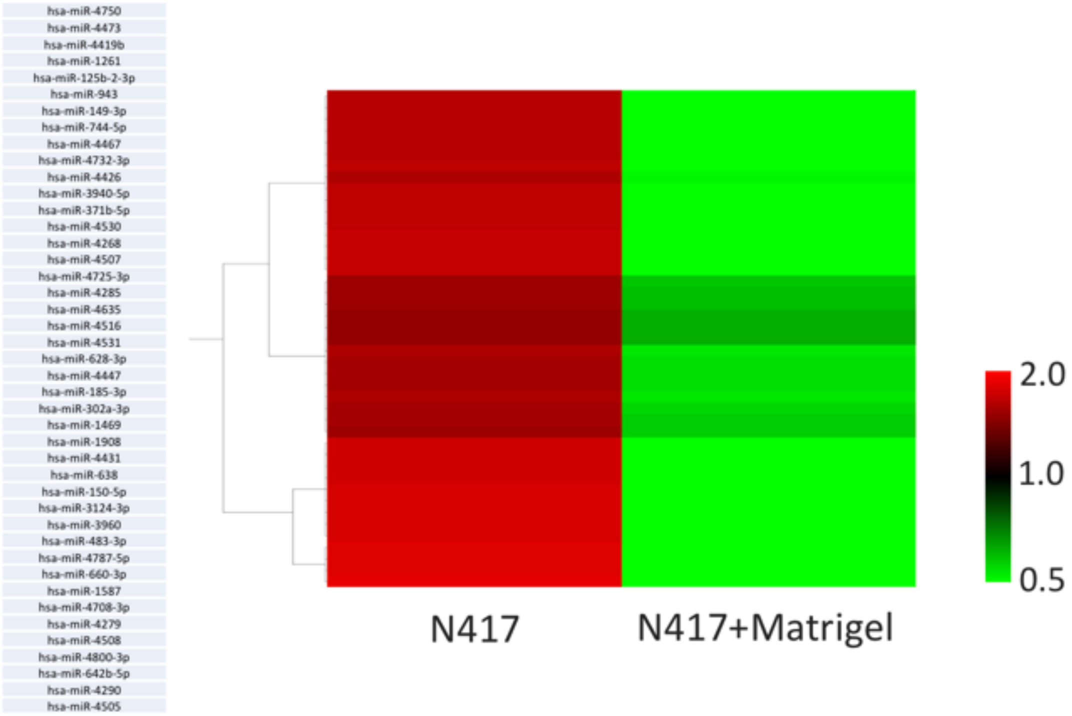

Extended Data Fig. 12. miRNA microarray data analysis. Heat map representing the relative miRNA expression levels in N417 cells treated with or without Matrigel. The miRNAs (43 miRNAs) that showed significant change were selected for hierarchical cluster analysis to generate the heat map. The color bar depicts the color contrast level of the heat map. Red and green indicate high and low expression levels, respectively.

Extended Data Figure 13

a

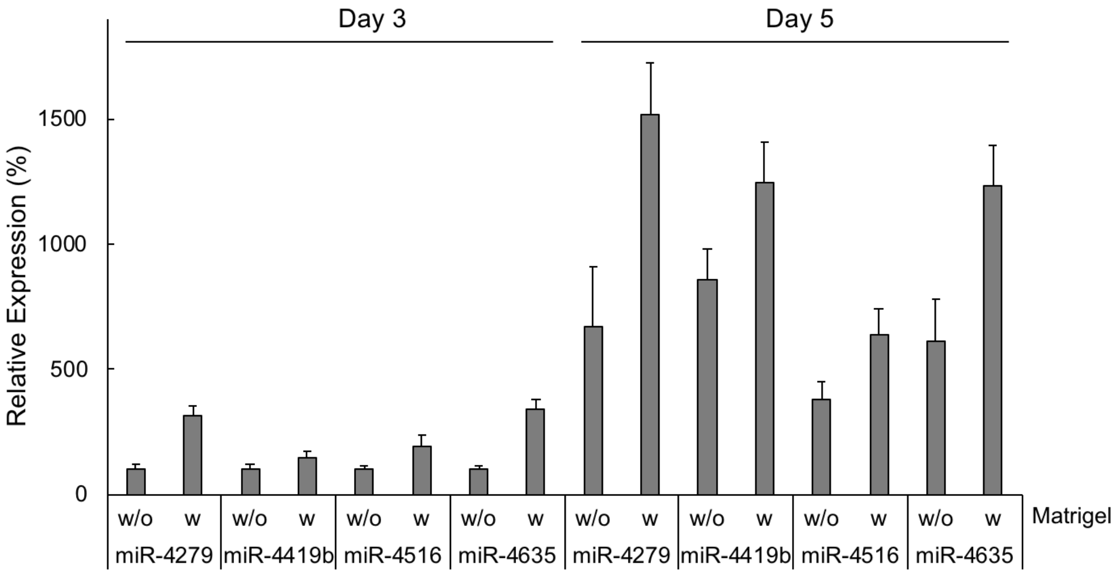

b

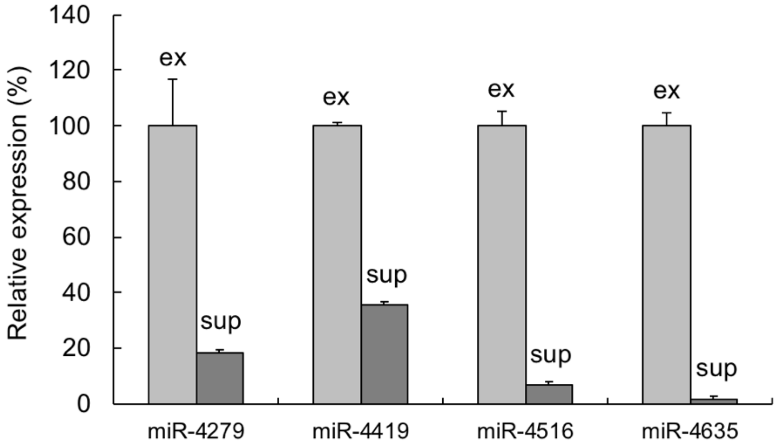

Extended Data Fig. 13. (**a**) Expression analysis of total miRNAs secreted in the culture medium. Total miRNA in the medium was prepared and each miRNA analyzed by qRT-PCR using specific primers. Data are mean ± S.D. (n=3) (**b**) Expression analysis of miRNAs incorporated in exosomes (ex) and not incorporated in exosome (sup) by qRT-PCR. Exosomes were prepared by ultracentrifugation and the resultant supernatant obtained. Total RNA in the exosome and supernatant fractions was prepared as described in Methods. Each miRNA expression is shown relative to its value in exosome (expressed as 100%). Data are mean ± S.D. (n=3)

Extended Data Figure 14

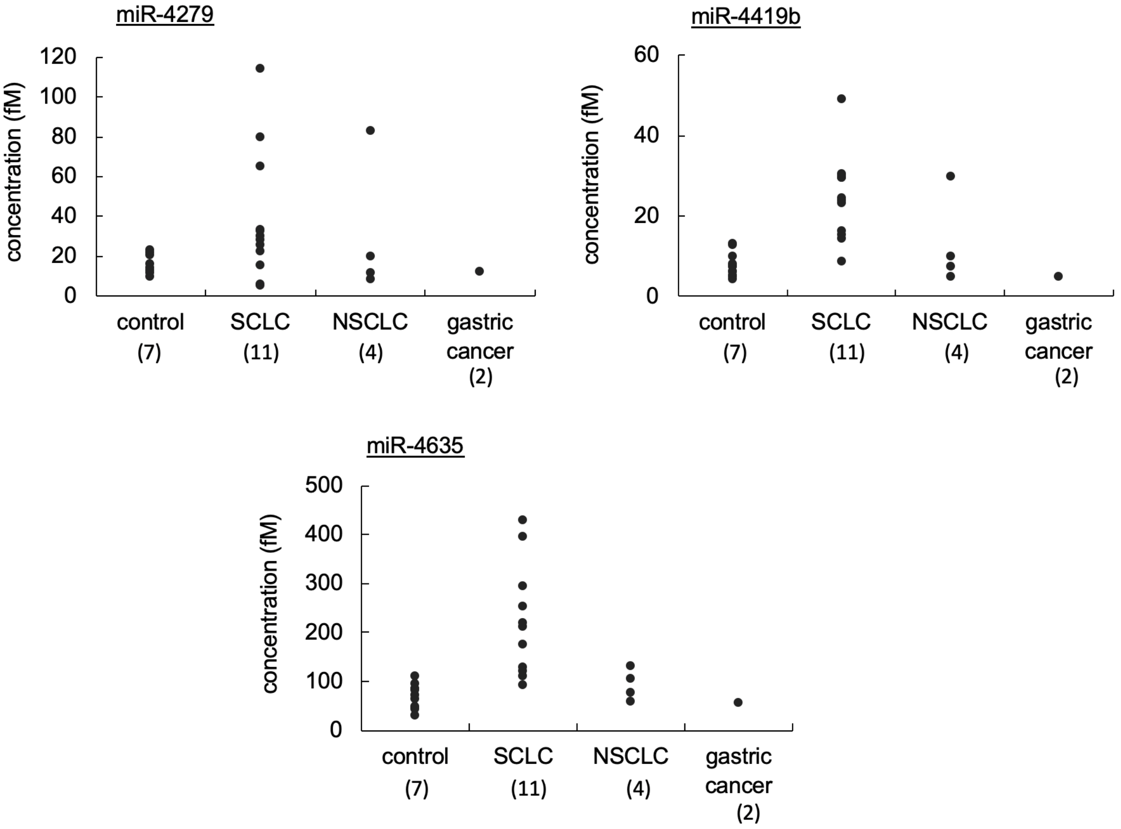

Extended Data Fig. 14. Analysis of miRNAs incorporated in exosome from cancer patient serum. Micro RNA in serum from various patients as well as healthy control donors was quantitated as described in Materials and Methods. Each miRNA（miR-4279, miR-4419b, miR-4635） was analyzed using specific primers as described. The numbers of samples from each disease are shown in parenthesis.

Extended Data Figure 15

Patients' characteristics

	sex	TNM	stage
SCLC	male	T4N3M1a	IV
SCLC	female	T4N3M1b	IV
SCLC	male	T4N1M0	IIIA
SCLC	male	T4N2M0	IIIB
SCLC	female	T4N3M1b	IV
SCLC	male	T4N3M0	IIIB
SCLC	male	T3N2M1a	IV
SCLC	male	T4N2M1b	IV
SCLC	male	T4N2MX	IIIB
SCLC	male	T4N0M1b	IV
SCLC	male	T4N3M1b	IV
NSCLC	female	T4N3M1b	IV
NSCLC	male	T2bN1M1b	IV
NSCLC	female	T4N2M0	IIIB
NSCLC	male	T2aN1M1a	IV
NSCLC	male	T3N3M1b	IV
